# Long-term risk of gynecologic malignancies in postmenopausal women with vaginal bleeding and benign endometrial lesions: a cohort study

**DOI:** 10.3389/fonc.2026.1859662

**Published:** 2026-07-20

**Authors:** Chunxiu Lv, Mingrui Su, Xiaojuan Zheng, Jinfeng Li, Lei Zhu, Shuzhen Wang, Jianxin Zhang

**Affiliations:** 1 Department of Obstetrics and Gynecology, Beijing Chaoyang Hospital Affiliated with Capital Medical University, Beijing, China; 2 Department of Obstetrics and Gynecology, Beijing Huairou Hospital, Beijing, China; 3 Department of Obstetrics and Gynecology, Xinhua Hospital, Beijing, China; 4 Department of Obstetrics and Gynecology, Beijing Maternal and Child Health Hospital, Beijing, China

**Keywords:** follow-up, gynecological malignancy, high risk, high-grade serous carcinoma, prognosis, postmenopausal bleeding

## Abstract

**Objective:**

This study aims to assess the long-term incidence risk of gynecologic malignancy in postmenopausal women presenting with vaginal bleeding and pathologically confirmed benign endometrial lesions, and to screen for clinical independent risk factors to provide evidence for risk stratification and follow-up management of this population.

**Methods:**

We conducted a retrospective cohort study of 696 postmenopausal women presenting with vaginal bleeding who underwent diagnostic endometrial curettage. Histopathological evaluation established the initial diagnosis; patients with initially benign endometrial pathology underwent short-term (3-month) histologic re-evaluation, and eligible individuals were enrolled in long-term clinical follow-up. We compared the distribution of pathological subtypes between initial and follow-up diagnoses and performed univariate Cox regression and multivariate Cox proportional hazards regression analyses to identify independent predictors of gynecologic malignancy.

**Results:**

The initial diagnosis rates of endometrial malignancy and precancerous lesions were 15.1% (105/696) and 2.9% (20/696), respectively. Among the 118 patients with initially benign endometrial pathology, 5.1% (6/118) and 7.6% (9/118) were diagnosed with malignancy and precancerous lesions, respectively, within 3 months of initial evaluation. Of the 453 patients eligible for long-term follow-up, 338 completed follow-up (25.4% lost to follow-up), during which 16 incident malignancies and 8 incident precancerous lesions were identified. High-grade serous carcinoma constituted a significantly higher proportion of malignancies detected during follow-up (43.8%) than of those diagnosed initially (6.5%; P < 0.001). Patients with high-grade serous carcinoma had a significantly lower BMI compared with those with endometrioid adenocarcinoma or precancerous lesions (P = 0.008); however, median diagnostic delay did not differ significantly across histologic subtypes (P = 0.850). Univariable Cox regression identified RDW, endometrial thickness, and age as factors associated with the risk of subsequent neoplasia. Multivariable Cox proportional hazards regression analysis confirmed baseline red blood cell distribution width (HR = 1.331, 95% CI: 1.086–1.630, P = 0.009) and endometrial thickness (HR = 4.452, 95% CI: 2.111–9.391, P < 0.001) as independent predictors of gynecologic malignancy.

**Conclusion:**

Our findings confirm that postmenopausal women with vaginal bleeding and initial benign endometrial lesions carry higher long-term gynecologic malignancy risk. Given their poor tumor prognosis, standardized screening and long-term surveillance are clinically vital for this high-risk cohort.

## 1 Introduction

Postmenopausal vaginal bleeding (PMB) is a prevalent clinical presentation, with an estimated prevalence of 4%–11% among postmenopausal women overalland up to 15% in those aged 65 years and older (1, 2). While the majority of cases are attributable to benign etiologies—including atrophic vaginitis, endometrial polyps, submucosal leiomyomas, and simple (non-atypical) endometrial hyperplasia—PMB remains a critical red flag for gynecologic malignancy, particularly endometrial carcinoma (3). Consequently, current national and international clinical practice guidelines universally recommend prompt and systematic evaluation of all postmenopausal women presenting with vaginal bleeding (4–7). All clearly state that any postmenopausal vaginal bleeding is considered “abnormal” and must undergo a systematic assessment, including transvaginal ultrasound measurement of endometrial thickness, diagnostic curettage, or endometrial biopsy under hysteroscopy, to rule out malignant lesions. This diagnostic and therapeutic paradigm has long centered on the “first bleeding”, emphasizing early identification and intervention, which has significantly improved the detection rate of endometrial cancer and the five-year survival rate.

However, in real clinical practice, a considerable number of patients may still experience recurrent or intermittent vaginal bleeding, known as “rebleeding”, after completing the initial assessment and excluding malignant lesions. These patients are often classified into the “benign follow-up group”, and there is a lack of unified standards for their subsequent management: some only undergo regular ultrasound examinations, some do not undergo further histological examinations, and some even interrupt follow-up on their own due to mild symptoms. It is worth noting that “rebleeding” is not a simple recurrent benign event; a few previous studies have suggested that it may be related to persistent endometrial microenvironment disorders, chronic inflammatory stimulation, improper use of hormone replacement therapy, or the progression of latent precancerous lesions. Especially when the bleeding intervals are prolonged, accompanied by new symptoms (such as discharge, lower abdominal distension), or when imaging indicates irregular endometrial thickening, the potential malignant risk cannot be ignored. Regrettably, the current clinical understanding of the long-term outcome of “rebleeding” is insufficient - there is a lack of systematic description of its natural course and quantitative assessment of the risk of malignant transformation over 10 years or more.

Several critical research gaps persist in the current literature. First, most cohort studies restrict follow-up to 3–5 years, limiting their ability to capture late-onset malignant transformation of indolent endometrial lesions—a key limitation given the typically protracted natural history of endometrial cancer (8). Second, high-quality long-term follow-up remains methodologically challenging due to substantial loss to follow-up (25.4% in this cohort), incomplete primary care documentation, and fragmented clinical data across healthcare institutions—factors that have undermined the external validity of numerous studies reporting follow-up rates below 70% (9). Notably, however, these suboptimal follow-up rates are not artifacts of poor study conduct but rather reflect the inherent constraints of real-world clinical practice within primary and regional healthcare settings. Rather than treating low follow-up as a flaw to be mitigated, this study intentionally incorporates it as a defining feature of its design: We conducted a retrospective cohort analysis of 696 consecutive patients with postmenopausal bleeding (PMB) admitted between 2011 and 2022, integrating longitudinal data from hospital electronic medical records to characterize the long-term oncologic outcomes among those with an initial benign endometrial histopathological diagnosis. Specifically, we investigated: (i) the temporal pattern of malignancy development; (ii) histologic progression—including transitions from atypical hyperplasia to endometrioid adenocarcinoma; (iii) the comprehensive profile of clinical and demographic risk factors (e.g., age at menopause, years since menopause, BMI, diabetes mellitus, and prior hormone therapy); and (iv) the differential risk of subsequent gynecologic malignancy associated with specific benign baseline diagnoses, including simple endometrial hyperplasia, endometrial polyps, and chronic endometritis (10).

The primary objective of this study is to clarify gynecological neoplasia in postmenopausal women with vaginal bleeding during long-term follow-up. The secondary objective is to assess the long-term risk of gynecological malignancies in such patients, thereby providing a basis for stratified management of postmenopausal women with vaginal bleeding, offering evidence-based support for the update of national and international clinical practice guidelines, and enhancing clinicians’ awareness of the risk of malignancy indicated by recurrent postmenopausal vaginal bleeding.

## 2 Methods

### 2.1 Study design

A retrospective cohort study was conducted using a cohort of women presenting with postmenopausal bleeding at Beijing Chaoyang Hospital Affiliated with Capital Medical University, a gynecologic oncology center in Beijing, China, between January 2011 and December 31, 2022. The primary outcome was the incidence of gynecologic malignancies or precancerous lesions. Study endpoints were the date of pathological diagnosis of gynecologic malignancies or precancerous lesions and the date of the last follow-up—December 31, 2024—for participants who had not been diagnosed with such conditions.

### 2.2 Study population and data collection

Inclusion criteria were: (1) postmenopausal status and (2) postmenopausal vaginal bleeding, confirmed by pelvic imaging, endometrial biopsy, and cervical cytology or biopsy. Exclusion criteria were: (1) prior hysterectomy; (2) active hemorrhagic or immune-mediated disorders; (3) synchronous malignancies in other organ systems; and (4) receipt of postmenopausal hormone therapy.

Data collected included baseline characteristics—namely, age, BMI, history of diabetes mellitus, history of hormone therapy, and transvaginal ultrasound–measured endometrial thickness.

All hematological parameters including red cell distribution width (RDW) were retrospectively retrieved from institutional electronic medical records. RDW data originated from routine preoperative complete blood count examinations performed within 24 hours prior to initial diagnostic curettage, with all blood samples analyzed using standardized clinical laboratory testing equipment of our hospital.

Pathological data: type of first biopsy pathology, final surgical pathology result (gold standard).
Follow-up data: follow-up time, type of new malignant tumor, occurrence time, treatment method.

### 2.3 Follow-up

All data were extracted from the electronic medical records database. Patients who met the inclusion criteria but not the exclusion criteria were enrolled. After evaluating women with PMB, eligibility was determined based on predefined inclusion and exclusion criteria; thereafter, clinical visit records, imaging results, and pathological findings were prospectively collected prior to ascertainment of the primary endpoint outcome. Recurrence of PMB—as well as associated symptoms including abnormal cervical screening results, abdominal discomfort, or atypical vaginal discharge—was systematically documented during subsequent monitoring.

During follow-up, women with indications for endometrial sampling or surgical intervention (laparoscopy or laparotomy) were managed by gynecologic oncologists and diagnosed by board-certified gynecologic pathologists. Clinical diagnosis and management were performed by gynecologic oncologists. Women without indications for biopsy were assigned to the observation group.

Indications for endometrial sampling included: (1) recurrent PMB; (2) prior abnormal endometrial imaging; and (3) abnormal cervical cytology. Indications for laparoscopic surgery or laparotomy in postmenopausal women with vaginal bleeding were: (1) suspected pelvic mass; (2) recurrent PMB despite normal hysteroscopic and colposcopic examinations; and (3) endometrial dysplasia identified on diagnostic curettage pathology.

Diagnoses of uterine fibroids and polyps were initially based on transvaginal ultrasonography findings during initial assessment. Final diagnoses were confirmed by histopathologic examination of surgical specimens obtained during the follow-up period.

### 2.4 Statistical analysis

Statistical analyses were performed using SPSS Statistics 27.0 (IBM Corp., Armonk, NY, USA), R version 3.6.1 (R Foundation for Statistical Computing, Vienna, Austria), and EmpowerStats (http://www.empowerstats.com; X&Y Solutions, Inc., Boston, MA, USA). For continuous variables, between-group comparisons were conducted using either the independent-samples t-test or the Wilcoxon rank-sum test, with test selection guided by formal assessment of normality (Shapiro–Wilk test) and homogeneity of variance (Levene’s test). Categorical variables were analyzed using Pearson’s chi-square test when all expected cell counts exceeded 5; otherwise, Fisher’s exact test was employed. The diagnostic miss rate was calculated from a 2×2 contingency table and evaluated via chi-square testing. To identify independent predictors of gynecologic malignancy, we first conducted univariable Cox regression, followed by multivariable Cox proportional hazards regression, adjusting for prespecified potential confounders—including age, BMI, diabetes mellitus, and hypertension, and so on.Cumulative incidence of gynecologic malignancy in women with postmenopausal vaginal bleeding was estimated using multivariable Cox regression, with time-to-event defined as the interval from initial benign histopathological diagnosis to diagnosis of malignancy (or last follow-up for censored cases). All statistical tests were two-sided, and a p-value < 0.05 was considered statistically significant. Cases with missing covariate data were excluded from the corresponding Cox regression analyses via complete-case analysis. Based on the regression coefficients of the final multivariable model, an individualized prognostic risk score was developed in accordance with the Transparent Reporting of a multivariable prediction model for Individual Prognosis Or Diagnosis (TRIPOD) guidelines (11).

## 3 Results

### 3.1 All cohort follow-up results

This retrospective study enrolled 696 postmenopausal women with vaginal bleeding. Following initial assessment, 105 patients were diagnosed with malignant tumors and 20 with precancerous lesions. Among the 20 patients initially diagnosed with precancerous lesions via endometrial biopsy, definitive surgical pathology (total hysterectomy and bilateral salpingo-oophorectomy) revealed 12 cases of malignancy and 8 of precancerous lesions. Within one month after diagnostic curettage, 58 patients with benign endometrial lesions underwent definitive surgery, histopathological examination confirmed 5 malignant tumors and 7 precancerous lesions. An additional 60 patients underwent definitive surgery within 3 months after initial assessment, yielding 1 malignant tumor and 2 precancerous lesions. Overall, the consistency analysis of the pathological results of diagnostic curettage and hysterectomy with bilateral salpingo-oophorectomy in postmenopausal women with vaginal bleeding shows that: (1) Among patients initially diagnosed with precancerous lesions of the endometrium, the rate of missed diagnosis of malignant tumors was 60.0% (12/20); (2) Among patients initially diagnosed with benign lesions of the endometrium, the rate of missed diagnosis of malignant tumors was 5.1% (6/118), and the rate of missed diagnosis of precancerous lesions was 7.6% (9/118).

**Figure 1:**
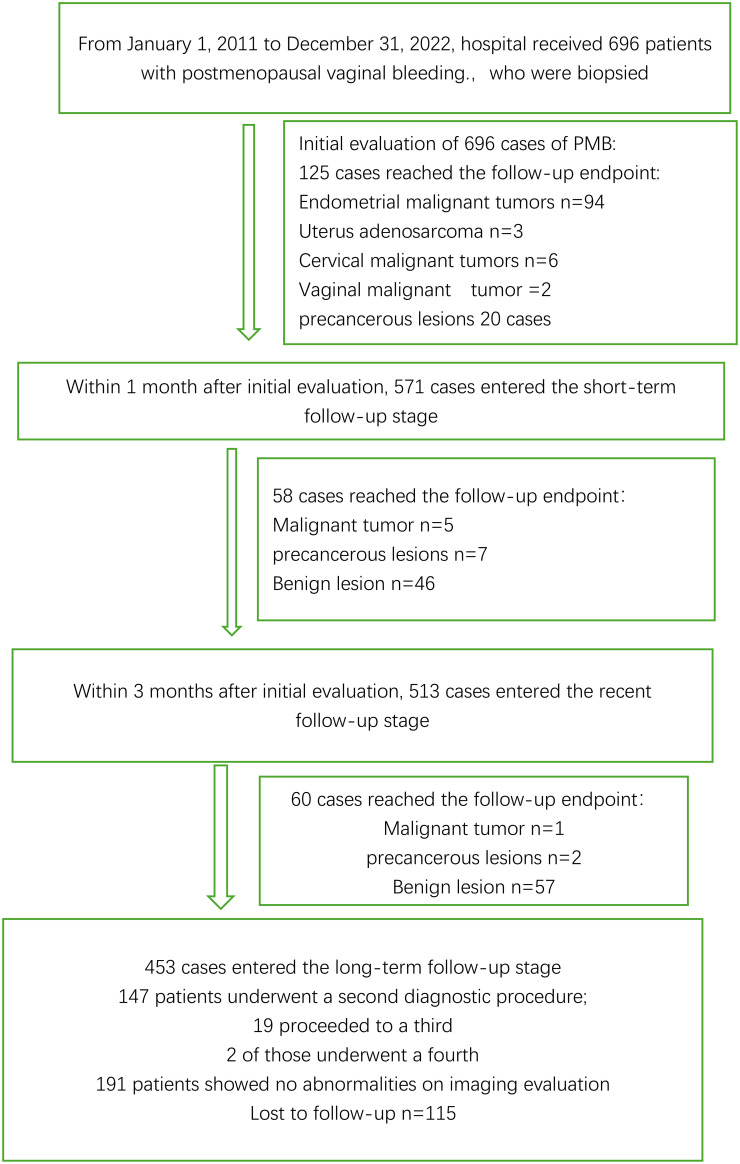
Screening process of the research subjects.

After the initial curettage and definitive surgical diagnosis, 453 patients entered long-term follow-up, with a predefined cut-off date of December 31, 2024. By this date, 338 patients provided valid follow-up data, while 115 were lost to follow-up (loss-to-follow-up rate: 115/453,25.4%). During the follow-up period, a total of 147 patients underwent second surgery, 19 patients underwent third surgery, and 4 patients underwent fourth surgery. A total of 16 cases of malignant tumors were diagnosed (12 during the second surgery, 3 during the third surgery, and 1 during the fourth surgery). At the end of the follow-up, a total of 8 cases of precancerous lesions were diagnosed.The remaining 191 patients showed no abnormalities on imaging evaluation (e.g., transvaginal ultrasound or pelvic MRI), fulfilling the study’s imaging-based follow-up endpoint criteria (**Figure 1**).

Clinical and pathological characteristics of patients diagnosed with malignant and precancerous lesions during long-term follow-up. A total of 453 people were included in the long-term follow-up, and 338 cases completed the follow-up. The general information of the followed women is shown in **Table 1**.

**Table 1:** Baseline clinical indicators in postmenopausal vaginal bleeding and confirmed to be benign at initial evaluation.

Pathology	Benign lesion	Precancerous lesion	Malignant disease	P-value
N	314	8	16	
Age	58.8 ± 7.8	60.1 ± 10.3	61.3 ± 0.1	0.427
<60 years	209 (66.6%)	6 (75.0%)	9 (56.2%)	0.606
≥60 years	105 (33.4%)	2 (25.0%)	7 (43.8%)	
Age at menopause	50.4 ± 3.5	52.0 ± 4.5	49.7 ± 3.3	0.312
Duration of menopause				0.399
< 5 years	203 (64.6%)	6 (75.0%)	8 (50.0%)	
≥5 years	111 (35.4%)	2 (25.0%)	8 (50.0%)	
BMI	25.7 ± 4.0	25.6 ± 3.5	27.7 ± 5.7	0.190
Smoking status N (%)				>0.05
Current	7 (2.28%)	1(12.5%)	2(12.5%)	
Never	307 (97.78%)	7(87.5%)	14(87.5%)	
Parity				0.654
Nulliparity	18 (5.73%)	2 (25.0%)	3(18.75%)	
1	230(73.25%)	5(62.5%)	9(56.25%)	
≥2	66 (21.02%)	1 (12.5%)	4(25%)	
Hypertension*				0.332
No	175 (55.9%)	5 (62.5%)	6 (37.5%)	
Yes	138 (44.1%)	3 (37.5%)	10 (62.5%)	
DM*				0.372
No	250 (80.1%)	8 (100.0%)	13 (81.2%)	
Yes	62 (19.9%)	0 (0.0%)	3 (18.8%)	
Previous history of malignant tumors*				0.710
No	273 (87.5%)	8 (100.0%)	14 (87.5%)	
Yes	39(12.5%)	0 (0.0%)	2 (12.5%)	
Family history of tumors*				0.450
Yes	45 (14.4%)	0 (0.0%)	3 (18.8%)	
No	267 (85.6%)	8 (100.0%)	13 (81.2%)	

BMI, Body Mass Index; DM, diabetes.

*Missing data were observed. One patient had missing data for hypertension; two patients each had missing data for diabetes mellitus, history of malignant tumors, and family history of cancer.

**Table 2** summarizes the long-term follow-up pathological outcomes among patients with initially benign endometrial lesions. Significant differences in clinical progression were observed across initial histopathological subtypes: endometrial hyperplasia demonstrated the highest rate of malignant transformation (5/29, 17.2%), followed by endometrial polyps (6/104, 5.8%); no malignant transformations occurred in proliferative endometrium and atrophic endometrium.

**Table 2:** Distribution of pathological diagnoses in patients undergoing long-term follow-up, stratified according to initial pathological diagnosis.

Initial pathological evaluation	Pathological diagnosis during the follow-up period							Imaging assessment	Total
	Malignant tumor	Precancerous lesion	Endometrial hyperplasia	Proliferative endometrium	Endometrial polyps	Atrophic endometrium	Endometritis		
Hyperplasia	5	3	5	6	2	2	4	2	29
Proliferative	0	1	1	18	3	5	6	9	43
Endometrial polyps	6	1	4	4	12	15	1	61	104
Atrophic	0	1	0	1	2	7	2	117	130
Endometritis	2	0	0	0	3	7	1	1	14
Failure to obtain tissue	3	2	1	1	0	9	1	1	18
Total	16	8	11	30	22	45	15	191	338

The distribution of histopathological diagnoses at initial presentation among patients with postmenopausal vaginal bleeding differs significantly from that of newly diagnosed gynecologic malignancies detected during long-term follow-up(Fisher X^2^ = 25.74, P = 0.000). Specifically, high-grade serous carcinoma accounted for 7 of 107 initial malignant diagnoses (6.5%), whereas it represented 7 of the 16 malignancies newly identified during follow-up (43.8%) (**Table 3**; **Supplementary Tables 1**, **2**). Among the 7 patients with high-grade serous papillary carcinoma, 3 had endometrial high-grade serous papillary carcinoma, 2 had endometrium combined with fallopian tube and ovary, and 2 had high-grade serous papillary carcinoma of the ovary.

**Table 3:** Outcomes of patients with initial endometrial pathological classification during follow-up.

Type	N	No neoplasia	High-grade serous adenocarcinoma	Endometrioid adenocarcinoma	Precancerous lesion	P
Pathology						
Proliferative	43	42 (97.67%)	0 (0.00%)	0 (0.00%)	1 (2.32%)	0.002
Atrophic	130	129 (99.23%)	0(0.00%)	0(0.00%%)	1(0.77%)	
Endometrial polyps&	104	97 (93.27%)	4 (3.86%)	2 (1.92%)	1 (0.96%)	
Hyperplasia	29	21 (72.41%)	1 (3.45%)	4 (13.79%)	3 (10.34%)	
Endometritis	14	12(85.71%)	0	2(14.29%)	0	
Failure to obtain tissue	18	13(72.22%)	2(11.11%)	1(5.56%)	2(11.11%)	
Others						
Endometrial thickness		0.54 ± 0.39	0.89 ± 0.57	0.91 ± 0.56	1.02 ± 0.98	<0.001
BMI		25.67 ± 4.04	23.83 ± 3.50	31.00 ± 5.18	25.60 ± 3.54	0.002
Total	338	314	7	9	8	

&Combined with submucosal uterine fibroids and endometrial polypsX2 = 25.87, P = 0.0021.

The overall follow-up duration ranged from 6 to 167 months (median, 69 months). Stratified by pathological outcome, follow-up durations were as follows: negative group (n = 314), 27.5–167 months (median, 68 months); The interval from the initial assessment to the confirmed diagnosis, high-grade serous papillary carcinoma (n = 7), 9–103 months (median, 49 months); endometrial adenocarcinoma (n = 9), 7–124 months (median, 53 months); and precancerous lesions (n = 8), 6–157 months (median, 63 months). One-way analysis of variance revealed no statistically significant difference in follow-up duration across the three pathological outcome groups (F = 0.164, P = 0.850).

Age and years since menopause were higher in the high-grade serous papillary carcinoma group (n = 7) compared with the endometrioid adenocarcinoma group (n = 9) and the precancerous lesion group (n = 8); however, these differences did not reach statistical significance. Patients with serous papillary carcinoma exhibited a significantly lower mean BMI (23.8; 95% CI, 20.6–27.1) than endometrioid adenocarcinoma with (31.0,95% CI, 24.6–40.6), and precancerous lesion (25.6,95%CI21.1-32.1), (F = 6.128, P = 0.008). No statistically significant differences were observed in other demographic characteristics, reproductive history, hematological parameters, or biochemical indicators.

### 3.2 Long-term risk assessment of incident gynecologic malignancies following postmenopausal vaginal bleeding

Univariate Cox regression analysis stratified by baseline endometrial pathology revealed that baseline age (HR = 1.10, 95% CI: 1.01–1.19; P = 0.006), baseline endometrial thickness (HR = 4.20, 95% CI: 1.70–10.30; P = 0.002), and red blood cell distribution width (RDW) (HR = 1.50, 95% CI: 1.10–2.00; P = 0.018) were risk factors for incident gynecological malignancies among postmenopausal women presenting with vaginal bleeding. Subgroup analyses demonstrated that baseline endometrial thickness remained significantly associated with long-term risk of gynecological malignancy across pathological subgroups—including endometrial polyps (HR = 1.30, 95% CI: 1.30–15.80; P = 0.016), atrophic endometrium or endometritis (HR = 12.50, 95% CI: 2.80–74.10; P = 0.027). In contrast, RDW showed a significant association with long-term malignancy risk exclusively in the endometrial hyperplasia subgroup (HR = 12.50, 95% CI: 1.30–117.10; P = 0.027) (**Supplementary Table 3**).

Univariable regression revealed that RDW, endometrial thickness and age were correlated with the risk of subsequent neoplasia. These variables were entered into the multivariable Cox model (**Table 4**) for further adjustment.

**Table 4:** Multivariate cox proportional hazards regression analysis to evaluate the risk of subsequent neoplastic development during long-term follow-up in patients with postmenopausal bleeding and histologically confirmed benign endometrial pathology.

	Coef.	SE	*z*	P	Hazard ratio (HR)	Hazard ratio, 95%CI	
RDW	0.2856	0.1035	2.7598	0.009	1.331	1.086	1.630
Endometrial thickness	1.4934	0.3808	3.9214	0.0001	4.452	2.111	9.391
Age	0.0418	0.0245	1.7030	0.0886	1.043	0.994	1.094

RDW, red blood cell distribution width; HR, hazard ratio; CI, confidence interval.

**Table 4** presents the results of multivariate Cox proportional hazards regression analysis for the risk of subsequent neoplastic progression among patients with postmenopausal bleeding and initially benign endometrial lesions, with three predictors including red blood cell distribution width (RDW), endometrial thickness, and age. Elevated RDW was an independent risk factor for subsequent neoplasia (HR = 1.331, 95%CI: 1.086–1.630, P = 0.009). Increased endometrial thickness also significantly predicted higher neoplastic risk (HR = 4.452, 95%CI: 2.111–9.391, P < 0.0001). By contrast, age showed no statistically significant independent predictive value (HR = 1.043, 95%CI: 0.994–1.094, P = 0.089).

**Figure 2:**
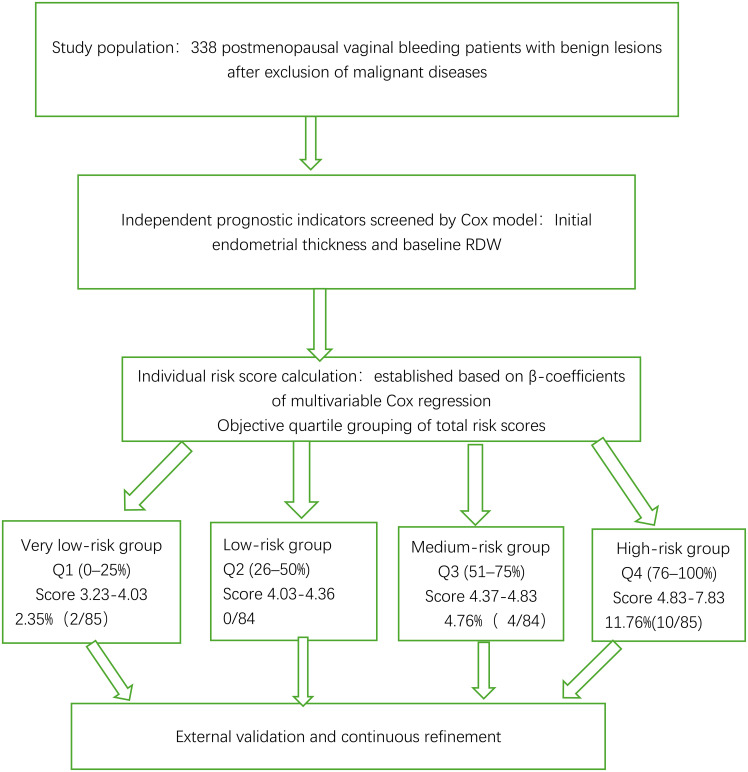
Kaplan–Meier survival curves for long-term genital tract malignancy risk in four-tier prognostic risk subgroups.

Based on the independent prognostic factors identified by multivariable Cox regression, including age, THINK, and RDW, an individualized prognostic risk score was constructed following the TRIPOD guidelines (11). All patients were stratified into four risk subgroups according to the quartile distribution of the continuous risk score. The cohort demonstrated a low overall event rate, but the four-tier risk stratification system effectively distinguished gradual differences in long-term malignant prognosis (Log-rank P = 0.046). Patients in the high-risk subgroup exhibited significantly inferior progression-free survival. The tumor transformation rate was 1.83% in the combined Q1+Q2 group, 4.7% in Q3, and 11.76% in Q4—demonstrating progressive risk stratification, robust discriminatory performance, and clinical utility of this objective algorithm (**Figure 2**; **Supplementary Figure 1**).

## 4 Discussion

A pivotal finding of this study was the marked enrichment of high-grade serous carcinoma (HGSC) among malignancies diagnosed during follow-up (43.8%) relative to those detected at baseline (6.5%; P < 0.001). This raises the critical question: do these follow-up diagnoses represent missed malignancies at initial evaluation, or truly *de novo* tumors? The long-term follow-up cohort comprised patients in whom malignancy and precancerous lesions had been rigorously excluded at baseline assessment. Patients who were missed during initial screening were retrospectively included in the baseline assessment Patients who were inadvertently included despite meeting prespecified exclusion criteria were identified during a 3-month post-baseline review and subsequently excluded.

Collectively, these findings strongly suggest that most follow-up-detected HGSC represents independent *de novo* pelvic malignancy rather than malignant transformation from preceding benign endometrial lesions diagnosed via initial blind diagnostic curettage. Critically, blind endometrial sampling cannot obtain tissue specimens from the fallopian tube or ovary, the primary anatomical origin of most high-grade serous carcinomas; accordingly, postmenopausal bleeding in these patients was mostly an indirect or secondary clinical manifestation instead of a consequence of primary endometrial disease.

In long-term follow-up, the rising incidence of HGSC observed during prolonged follow-up is consistent with the inherent biological characteristics of this aggressive tumor subtype. Overall, HGSC accounted for11.4% (14/123), of all incident malignancies in our cohort, which is in line with previously published epidemiological data (8). Notably, even after ruling out definite endometrial carcinoma and premalignant lesions at baseline, patients with postmenopausal bleeding still carry persistent long-term risk of subsequent HGSC. Therefore, long-term surveillance for high-risk individuals (advanced age, low BMI, elevated RDW) should incorporate regular pelvic imaging (ultrasound or MRI for adnexal assessment) alongside repeated endometrial sampling, rather than relying solely on sequential endometrial curettage.

TP53 mutation (90%+) is the core mechanism of high-grade serous carcinoma of the uterus, fallopian tubes and ovaries (12, 13). After mutation, DNA damage cannot be repaired and cell apoptosis is blocked. The molecular mechanisms of carcinogenesis in the three are similar, but the cells of origin are different (14, 15). Single-cell clones with p53 mutations first appear in the atrophied endometrium, gradually developing into carcinoma *in situ* (EIC), and then infiltrating into high-grade serous papillary carcinoma, ovarian HGSC most commonly originates from the fallopian tube epithelium, with secretory cell outgrowths (STIC) capable of undergoing occult progression in the pelvic cavity for 3–5 years prior to development of clinically apparent carcinoma (16, 17). This study followed up for 2 to 14 years. Seven new cases of high-grade papillary-lobular carcinoma were found, distributed from nearly 1 year to 9 years, with no obvious time clustering phenomenon.

To sum up,”time-dependent new malignant transformation/hidden precancerous lesion progression”: At initial diagnosis, no cancer focus or precancerous lesion is identified (or it is occult and undetectable). With increasing duration of postmenopausal time, under the combined effects of genetic drivers, microenvironmental remodeling, and epithelial metaplasia, such lesions gradually emerge and progress to high-grade serous carcinoma (HGSC). Thus, the observed increase in incidence during follow-up reflects the cumulative occurrence of malignancies—not delayed diagnosis at baseline.

This study reveals a substantial early false-negative rate—12.7% within 3 months following an initial histopathologically “benign” endometrial curettage—in patients who, despite negative diagnostic findings on first-line sampling, were later diagnosed with occult endometrial carcinoma or precancerous lesions (5.1% and 7.6%, respectively) at the time of definitive surgery (hysterectomy with bilateral salpingo-oophorectomy). This combined rate underscores the intrinsic limitation of blind curettage: its suboptimal sensitivity compared with hysteroscopy-guided, site-specific biopsy. Drawing on our longitudinal cohort (2011–2022), we advocate for hysteroscopy-guided endometrial sampling as the recommended initial diagnostic standard in the evaluation of postmenopausal bleeding (PMB), to mitigate avoidable diagnostic delays and improve early detection.

Another important finding of this study is that RDW is associated with the occurrence of long-term gynecological malignant tumors. Elevated RDW was independently associated with long-term malignancy risk (HR = 1.331, P = 0.009) among PMB patients in our cohort, which could be biologically explained by two well-established mechanisms supported by previous evidence. First, RDW serves as a reliable peripheral surrogate marker for chronic low-grade systemic inflammation, which persistently facilitates oncogenic transformation of pelvic epithelial tissues via pro-inflammatory cytokines and disordered tumor microenvironment regulation; multiple long-term population cohort studies verified that elevated baseline RDW predicts subsequent incident endometrial and ovarian malignancies in postmenopausal women, consistent with our real-world follow-up findings (18, 19). Second, persistent occult genital bleeding derived from underlying benign endometrial lesions leads to subclinical iron deficiency, disrupting normal erythropoiesis and increasing RDW concentration; such repeated chronic mucosal injury also gradually elevates latent malignant transformation risk over years of surveillance (20, 21) For HGSC specifically, cumulative evidence confirmed that raised pretreatment RDW reflects excessive systemic inflammatory burden, an important promoting factor for the initiation and progression of tubal-origin high-grade serous carcinoma (22, 23).

Univariate regression analysis identified three predictors of endometrial malignancy: age at presentation, transvaginal ultrasound–measured endometrial thickness, and red blood cell distribution width (RDW). Multivariate Cox proportional hazards regression demonstrated that elevated RDW and increased endometrial thickness were independent risk factors for subsequent neoplastic development in patients with postmenopausal bleeding and benign initial endometrial lesions,. Age was not an independent prognostic factor with no statistical significance.

Endometrial thickness emerged as a validated imaging-based predictor (HR = 4.452; P = 0.0001), reinforcing its utility as a first-line triage tool. Most notably, RDW—traditionally associated with anemia and chronic inflammation—was revealed as a novel, statistically significant predictor. Its elevation may reflect subclinical inflammatory processes or iron-deficiency sequelae linked to occult endometrial neoplasia. Elevated RDW may enhance oxidative stress, leading to DNA base oxidative damage and increasing genomic instability.The relationship between RDW and gastrointestinal tracttumors has been reported, when the RDW of colorectal cancer patients is greater than 14.1%, the tumor stage is more advanced,the lymphatic metastasis and infiltration depth were both significantly increased (24–26). There are no reports of relationship with gynecological malignant tumors has not been reported. Studies show that RDW is a prognostic factor for melanoma (27–29).

Several limitations warrant acknowledgment. First, as a single-center retrospective study, this analysis is susceptible to selection bias; therefore, external validation through multicenter prospective cohort studies is urgently needed, such studies should have a larger sample size and a more diverse range of demographic and clinical characteristics. Secondly, the 25.4% loss to follow-up rate may affect the robustness of the research results. Notably, there is a significant difference in the distribution of endometrial pathological diagnoses between the lost-to-follow-up patients and those who completed the baseline assessment (see Appendix 2): although the proportion of proliferative endometrium or atrophic endometrium is higher in the lost-to-follow-up group, these two types of women have the lowest proportion of new malignant tumors in the long-term follow-up, but the proportion of lost-to-follow-up patients is high among those with endometritis, endometrial polyps, and those insufficient endometrial tissue,which the proportion of these three types of pathological conditions among newly developed malignant tumors,9/16. This non-random loss to follow-up may introduce a certain bias and thereby affect the credibility of the results. Thirdly, several potential confounding factors - such as hormone replacement therapy and germline or somatic gene mutations, were not systematically evaluated. Finally, the prognostic value of the predictive factors identified in this study has not been verified, and this should be a key focus of future research.

To overcome these limitations, we propose conducting a bidirectional, multicenter cohort study to validate and refine the risk stratification framework. We will integrate existing real-world cohort data with prospective, multicenter population data to develop next-generation quantitative prediction models. The independent risk factors identified in this study, along with their regression coefficients, demonstrate significant prognostic value (**Figure 2**). These findings support the further development of a scalable, dynamically updated predictive tool for precise risk management. Concurrently, mechanistic studies will be initiated to elucidate the molecular pathways through which independent risk markers drive long-term tumorigenesis. Additionally, a prospective intervention trial will be conducted to evaluate the clinical utility of risk-stratified monitoring strategies for PMB.

## 5 Conclusion

Despite these limitations, our study provides important clinical insights. This is the first time it has been clearly demonstrated that among postmenopausal women with vaginal bleeding, the proportion of HGSC confirmed by follow-up is much higher than the initial diagnosis. It was found that low BMI is a characteristic biomarker of HGSC in the population of postmenopausal vaginal bleeding, revealing the pathogenic difference between HGSC and endometrioid adenocarcinoma in terms of estrogen dependence. For the first time, RDW was identified as an independent predictor of endometrial malignant lesions in postmenopausal women with vaginal bleeding. Combined endometrial thickness, a simple and practical risk prediction model can be constructed, providing a new screening solution for primary care or resource-limited medical institutions.

## Data Availability

The original contributions presented in the study are included in the article/**Supplementary Material**. Further inquiries can be directed to the corresponding authors.
