# Supplementary material for: Long-term risk of gynecologic malignancies in postmenopausal women with vaginal bleeding and benign endometrial lesions: a cohort study
Source: Front Oncol. 2026 Jul 20;16:1859662. doi: 10.3389/fonc.2026.1859662 (PMC13430443; doi:10.3389/fonc.2026.1859662)
Supplement: Supplementary file 1 [file Table1.docx]

S-table-2 Distribution of baseline pathological types in patients lost to follow-up and those who completed follow-up

| Initial pathological classification | Follow-up | Lost follow up | Total |
| --- | --- | --- | --- |
| Endometrial hyperplasia | 29 | 2 | 31 |
| Proliferative endometrium | 43 | 22 | 65 |
| Endometrial polyps | 104 | 32 | 136 |
| Atrophic endometrium | 130 | 31 | 161 |
| Endometritis | 14 | 10 | 24 |
| Failure of biopsy sampling | 18 | 18 | 36 |
| Total | 338 | 115 | 453 |

X^2^=23.87,P=0.000

S-table-1 Composition of pathological classification at each diagnostic stage in long-term follow-up of menopausal vaginal bleeding patients

|  | Endometrioid adenocarcinoma | Type 2 endometrial cancer and HGSC | others | Precancerous lesion |
| --- | --- | --- | --- | --- |
| The initial assessment | 100 | 7 | 10 | 8 |
| Recently follow-up | 6 | 0 | 0 | 9 |
| Long-term follow up | 9 | 7 | 0 | 8 |

Fisher X2=33.131，P=0.000

Merging other subtypes and endometrioid adenocarcinoma; merging recent follow-up and initial assessment
