# Supplementary material for: Long-term risk of gynecologic malignancies in postmenopausal women with vaginal bleeding and benign endometrial lesions: a cohort study
Source: Front Oncol. 2026 Jul 20;16:1859662. doi: 10.3389/fonc.2026.1859662 (PMC13430443; doi:10.3389/fonc.2026.1859662)
Supplement: Supplementary file 2 [file Table2.docx]

S-table-3 Results of the univariate analysis of gynecologic malignancies following postmenopausal vaginal bleeding

| Variables | Statistics | LASTPATHOLIGY |
| --- | --- | --- |
| AGE | 59.0 ± 7.9 | 1.1 (1.0, 1.1) 0.006 |
| BMI | 25.8 ± 4.1 | 1.0 (0.9, 1.1) 0.578 |
| THINK | 0.6 ± 0.4 | 4.2 (1.7, 10.3) 0.002 |
| Atrophic endometrium | 0.4 ± 0.4 | 13.9 (2.7, 70.0) 0.001 |
| Endometrial polyps | 0.7 ± 0.4 | 4.6 (1.3, 15.8) 0.016 |
|  |  |  |
| RDW | 12.7 ± 1.3 | 1.5 (1.1, 2.0) 0.018 |
| Endometrial hyperplasia | 12.7 ± 1.1 | 12.5 (1.3, 117.0) 0.027 |
