# Supplementary material for: Long-term risk of gynecologic malignancies in postmenopausal women with vaginal bleeding and benign endometrial lesions: a cohort study
Source: Front Oncol. 2026 Jul 20;16:1859662. doi: 10.3389/fonc.2026.1859662 (PMC13430443; doi:10.3389/fonc.2026.1859662)
Supplement: Supplementary file 3 [file DataSheet1.pdf]

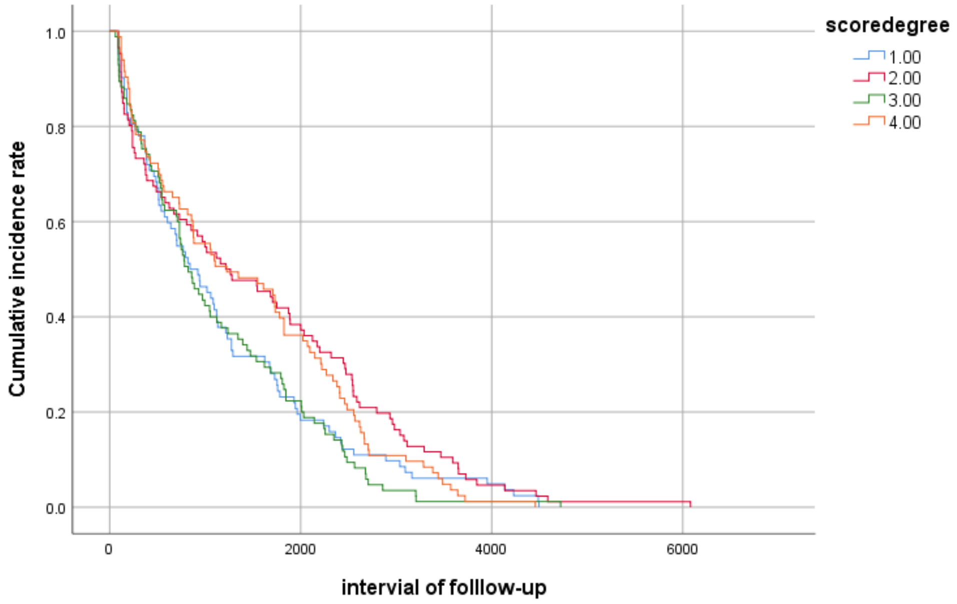

Figure S1. Kaplan–Meier survival curves for long-term genital tract malignancy risk in four-tier prognostic risk subgroups

Log-rank test, P = 0.046. Four-tier risk stratification was defined by the quartile values of the individualized Cox regression-based prognostic score
